# 2-(1*H*-Imidazol-1-yl)-4,6-dimethyl­pyrimidine

**DOI:** 10.1107/S1600536811044357

**Published:** 2011-10-29

**Authors:** Dao-Hui Yu, Jie-Ying Wu

**Affiliations:** a Department of Chemistry, Anhui University, Hefei 230039, People’s Republic of China, and Key Laboratory of Functional Inorganic Materials Chemistry, Hefei 230039, People’s Republic of China

## Abstract

The asymmetric unit of the title compound, C_9_H_10_N_4_, consists of two mol­ecules in which the dihedral angles between the planes of the imidazole and pyrimidine rings are 4.8 (1) and 2.1 (1)°.

## Related literature

For related pyrimidine derivatives, see: Wu *et al.* (2008 ▶); Cetina *et al.* (2005 ▶); Liu *et al.* (2007 ▶).
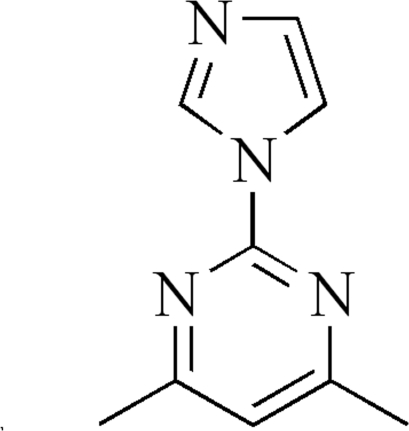


## Experimental

### 

#### Crystal data

C_9_H_10_N_4_
*M* *_r_* = 174.21
Monoclinic, 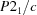

*a* = 9.304 (5) Å
*b* = 26.756 (5) Å
*c* = 7.129 (5) Å
β = 91.259 (5)°
*V* = 1774.2 (16) Å^3^
*Z* = 8
Mo *K*α radiation
μ = 0.09 mm^−1^
*T* = 296 K
0.20 × 0.10 × 0.10 mm

#### Data collection

Bruker SMART diffractometer
11971 measured reflections
3091 independent reflections
2365 reflections with *I* > 2σ(*I*)
*R*_int_ = 0.040

#### Refinement

*R*[*F* ^2^ > 2σ(*F* ^2^)] = 0.064
*wR*(*F* ^2^) = 0.197
*S* = 1.14
3091 reflections
240 parameters
H-atom parameters constrained
Δρ_max_ = 0.25 e Å^−3^
Δρ_min_ = −0.19 e Å^−3^

### 

Data collection: *SMART* (Bruker, 2002 ▶); cell refinement: *SAINT* (Bruker, 2002 ▶); data reduction: *SAINT*; program(s) used to solve structure: *SHELXS97* (Sheldrick, 2008 ▶); program(s) used to refine structure: *SHELXL97* (Sheldrick, 2008 ▶); molecular graphics: *SHELXTL* (Sheldrick, 2008 ▶); software used to prepare material for publication: *SHELXTL*.

## Supplementary Material

Crystal structure: contains datablock(s) I, global. DOI: 10.1107/S1600536811044357/ng5247sup1.cif

Structure factors: contains datablock(s) I. DOI: 10.1107/S1600536811044357/ng5247Isup2.hkl

Supplementary material file. DOI: 10.1107/S1600536811044357/ng5247Isup3.cml

Additional supplementary materials:  crystallographic information; 3D view; checkCIF report

## Figures and Tables

**Fig. 1.**
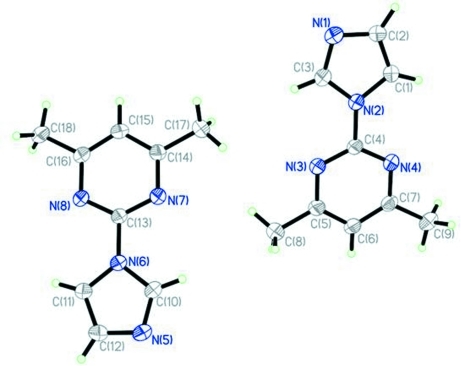
The molecular structure of the title molecule(I) showing 30% probability displacement ellipsoids.

**Fig. 2.**
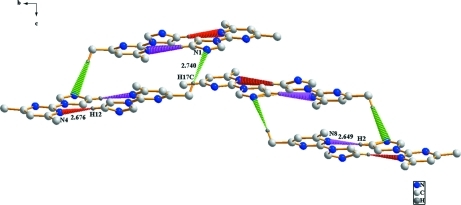
The packing diagram of the title compound(I). H atoms not involved in hydrogen bonds are omitted.

## supplementary crystallographic information

### Comment

In recent years,pyrimidine-based materials had been widely investigated for their high electron affinity in the area of nonlinear optical materials (Liu *et al.*, 2007); the crystal structures of a small number of pyrimidine derivatives have been widely reported (Wu *et al.*, 2008; Cetina *et al.*, 2005). The title molecule is a contribution to this topic. In (I), (Fig.1), the dihedral angles between imidazole and pyrimidine rings of the two molecules are only 4.8 (1) and 2.1 (1) °, which indicate that the molecules are almost co-planar. In the crystal structure, neighboring molecules are connected through weak intermolecular C—H···N interactions (Fig. 2).

### Experimental

CuI (0.19 g,1 mmol), 1,10-phenanthroline (0.6 g,3 mmol) and DMF (5 ml) were added to a three-necked flask equipped with a magnetic stirrer and a reflux condenser. The reaction mixture turned brown and was kept stirred for 5 min. Then, *t*-BuOK (1.12 g,10 mmol), imidazole (1.36 g, 20 mmol), 2-iodo-4,6-dimethylpyrimidine (0.46 g, 2 mmol) and a catalytic amount of 18-crown-6 were added in sequentially. After complete addition, the mixture was heated under nitrogen for about 2 h, then cooled to room temperature. The residue was extracted with 200 ml of dichloromethane, washed four times with distilled water, and dried with anhydrous MgSO_4_. Then it was filtered and concentrated and purified by flash column- chromatography on silica. Elution with petroleum/ethyl acetate (2:1) gave colorless crystals. Yield: 0.2 g (60%).

### Refinement

All hydrogen atoms were placed in geometrically idealized positions and constrained to ride on their parent atoms with C—H = 0.93–0.96 Å, *U*_iso_(H) = 1.2 - 1.5*U*_eq_(C).

### Crystal data

**Table:**

C_9_H_10_N_4_	*F*(000) = 736
*M**_r_* = 174.21	*D*_x_ = 1.304 Mg m^−^^3^
Monoclinic, *P*2_1_/*c*	Mo *K*α radiation, λ = 0.71069 Å
Hall symbol: -P 2ybc	Cell parameters from 3786 reflections
*a* = 9.304 (5) Å	θ = 2.7–25.4°
*b* = 26.756 (5) Å	µ = 0.09 mm^−^^1^
*c* = 7.129 (5) Å	*T* = 296 K
β = 91.259 (5)°	Needle, white
*V* = 1774.2 (16) Å^3^	0.20 × 0.10 × 0.10 mm
*Z* = 8	

### Data collection

**Table:**

Bruker SMART diffractometer	2365 reflections with *I* > 2σ(*I*)
Radiation source: sealed tube	*R*_int_ = 0.040
graphite	θ_max_ = 25.0°, θ_min_ = 1.5°
ω scans	*h* = −11→10
11971 measured reflections	*k* = −26→31
3091 independent reflections	*l* = −8→8

### Refinement

**Table:**

Refinement on *F*^2^	Secondary atom site location: difference Fourier map
Least-squares matrix: full	Hydrogen site location: inferred from neighbouring sites
*R*[*F*^2^ > 2σ(*F*^2^)] = 0.064	H-atom parameters constrained
*wR*(*F*^2^) = 0.197	*w* = 1/[σ^2^(*F*_o_^2^) + (0.0895*P*)^2^ + 0.9302*P*] where *P* = (*F*_o_^2^ + 2*F*_c_^2^)/3
*S* = 1.14	(Δ/σ)_max_ < 0.001
3091 reflections	Δρ_max_ = 0.25 e Å^−^^3^
240 parameters	Δρ_min_ = −0.19 e Å^−^^3^
0 restraints	Extinction correction: *SHELXL97* (Sheldrick, 2008), Fc^*^=kFc[1+0.001xFc^2^λ^3^/sin(2θ)]^-1/4^
Primary atom site location: structure-invariant direct methods	Extinction coefficient: 0.008 (2)

### Special details

**Table:**

Geometry. All e.s.d.'s (except the e.s.d. in the dihedral angle between two l.s. planes) are estimated using the full covariance matrix. The cell e.s.d.'s are taken into account individually in the estimation of e.s.d.'s in distances, angles and torsion angles; correlations between e.s.d.'s in cell parameters are only used when they are defined by crystal symmetry. An approximate (isotropic) treatment of cell e.s.d.'s is used for estimating e.s.d.'s involving l.s. planes.
Refinement. Refinement of *F*^2^ against ALL reflections. The weighted *R*-factor *wR* and goodness of fit *S* are based on *F*^2^, conventional *R*-factors *R* are based on *F*, with *F* set to zero for negative *F*^2^. The threshold expression of *F*^2^ > σ(*F*^2^) is used only for calculating *R*-factors(gt) *etc*. and is not relevant to the choice of reflections for refinement. *R*-factors based on *F*^2^ are statistically about twice as large as those based on *F*, and *R*- factors based on ALL data will be even larger.

### Fractional atomic coordinates and isotropic or equivalent isotropic displacement parameters (Å^2^)

**Table:**

	*x*	*y*	*z*	*U*_iso_*/*U*_eq_	
N7	0.3875 (2)	0.45208 (8)	0.7788 (3)	0.0472 (6)	
N2	0.1912 (2)	0.21267 (8)	0.6762 (3)	0.0493 (6)	
N4	−0.0469 (2)	0.22566 (9)	0.5881 (3)	0.0483 (6)	
N8	0.5461 (2)	0.52131 (8)	0.7336 (3)	0.0461 (6)	
N6	0.3095 (2)	0.53376 (8)	0.8162 (3)	0.0465 (6)	
C13	0.4215 (3)	0.50025 (10)	0.7736 (4)	0.0420 (6)	
C14	0.4946 (3)	0.42061 (10)	0.7404 (4)	0.0471 (7)	
N3	0.1114 (2)	0.29410 (9)	0.6535 (3)	0.0513 (6)	
C15	0.6291 (3)	0.43849 (11)	0.6982 (4)	0.0517 (7)	
H15	0.7034	0.4164	0.6727	0.062*	
C4	0.0780 (3)	0.24630 (10)	0.6363 (4)	0.0453 (7)	
N5	0.0973 (3)	0.56080 (10)	0.9067 (4)	0.0649 (8)	
C5	0.0032 (3)	0.32600 (11)	0.6131 (4)	0.0539 (8)	
C16	0.6525 (3)	0.48921 (11)	0.6942 (4)	0.0474 (7)	
C7	−0.1541 (3)	0.25785 (11)	0.5495 (4)	0.0494 (7)	
C6	−0.1306 (3)	0.30860 (11)	0.5581 (4)	0.0550 (8)	
H6	−0.2041	0.3309	0.5271	0.066*	
C10	0.1743 (3)	0.52165 (12)	0.8750 (4)	0.0552 (8)	
H10	0.1420	0.4890	0.8903	0.066*	
N1	0.4063 (3)	0.18529 (10)	0.7662 (5)	0.0728 (9)	
C11	0.3144 (3)	0.58522 (11)	0.8102 (4)	0.0548 (8)	
H11	0.3914	0.6051	0.7755	0.066*	
C9	−0.2980 (3)	0.23589 (13)	0.4997 (5)	0.0647 (9)	
H9A	−0.2873	0.2114	0.4027	0.097*	
H9B	−0.3615	0.2619	0.4560	0.097*	
H9C	−0.3373	0.2203	0.6086	0.097*	
C3	0.3271 (3)	0.22439 (12)	0.7368 (5)	0.0622 (9)	
H3	0.3593	0.2570	0.7551	0.075*	
C18	0.7944 (3)	0.51142 (13)	0.6463 (5)	0.0625 (9)	
H18A	0.7812	0.5352	0.5464	0.094*	
H18B	0.8582	0.4854	0.6070	0.094*	
H18C	0.8351	0.5279	0.7546	0.094*	
C17	0.4608 (4)	0.36581 (11)	0.7441 (5)	0.0664 (9)	
H17A	0.3708	0.3607	0.8044	0.100*	
H17B	0.5356	0.3484	0.8123	0.100*	
H17C	0.4545	0.3533	0.6180	0.100*	
C1	0.1867 (3)	0.16122 (11)	0.6687 (5)	0.0629 (9)	
H1	0.1088	0.1413	0.6328	0.076*	
C12	0.1844 (3)	0.60062 (12)	0.8651 (5)	0.0647 (9)	
H12	0.1569	0.6339	0.8738	0.078*	
C8	0.0358 (4)	0.38062 (12)	0.6319 (6)	0.0768 (11)	
H8A	0.0052	0.3923	0.7520	0.115*	
H8B	−0.0143	0.3987	0.5344	0.115*	
H8C	0.1374	0.3859	0.6214	0.115*	
C2	0.3180 (3)	0.14584 (13)	0.7239 (6)	0.0734 (10)	
H2	0.3456	0.1125	0.7323	0.088*	

### Atomic displacement parameters (Å^2^)

**Table:**

	*U*^11^	*U*^22^	*U*^33^	*U*^12^	*U*^13^	*U*^23^
N7	0.0400 (13)	0.0420 (13)	0.0596 (14)	−0.0005 (10)	−0.0030 (10)	−0.0009 (10)
N2	0.0380 (13)	0.0443 (14)	0.0659 (15)	−0.0011 (10)	0.0068 (11)	0.0059 (11)
N4	0.0401 (13)	0.0492 (14)	0.0559 (14)	−0.0035 (10)	0.0065 (10)	0.0015 (10)
N8	0.0342 (12)	0.0457 (14)	0.0583 (14)	−0.0013 (10)	−0.0026 (10)	−0.0007 (10)
N6	0.0354 (12)	0.0439 (13)	0.0601 (14)	0.0023 (10)	−0.0018 (10)	−0.0037 (10)
C13	0.0358 (14)	0.0414 (15)	0.0485 (15)	0.0028 (11)	−0.0041 (11)	−0.0017 (11)
C14	0.0433 (15)	0.0424 (15)	0.0553 (16)	0.0035 (12)	−0.0057 (12)	−0.0024 (12)
N3	0.0425 (13)	0.0436 (14)	0.0681 (15)	−0.0002 (11)	0.0125 (11)	0.0031 (11)
C15	0.0395 (15)	0.0509 (18)	0.0645 (18)	0.0094 (13)	−0.0011 (13)	−0.0025 (13)
C4	0.0387 (15)	0.0454 (16)	0.0522 (16)	0.0021 (12)	0.0109 (12)	0.0040 (12)
N5	0.0360 (13)	0.0625 (17)	0.096 (2)	0.0053 (12)	0.0027 (13)	−0.0125 (14)
C5	0.0479 (17)	0.0472 (17)	0.0672 (18)	0.0042 (13)	0.0165 (14)	0.0047 (13)
C16	0.0366 (14)	0.0505 (17)	0.0548 (17)	0.0018 (12)	−0.0038 (12)	0.0009 (12)
C7	0.0404 (15)	0.0578 (18)	0.0504 (16)	0.0026 (13)	0.0092 (12)	0.0050 (13)
C6	0.0411 (16)	0.0547 (19)	0.0694 (19)	0.0047 (13)	0.0094 (14)	0.0074 (14)
C10	0.0357 (15)	0.0547 (18)	0.075 (2)	−0.0004 (13)	0.0035 (13)	−0.0041 (14)
N1	0.0422 (15)	0.0608 (18)	0.115 (2)	0.0029 (13)	0.0018 (15)	0.0157 (16)
C11	0.0427 (16)	0.0431 (16)	0.078 (2)	0.0040 (13)	0.0000 (14)	−0.0005 (14)
C9	0.0428 (17)	0.081 (2)	0.071 (2)	−0.0022 (16)	0.0011 (14)	−0.0004 (17)
C3	0.0402 (17)	0.0538 (19)	0.093 (2)	−0.0027 (14)	−0.0002 (15)	0.0081 (16)
C18	0.0376 (16)	0.064 (2)	0.086 (2)	0.0005 (14)	0.0039 (15)	0.0028 (16)
C17	0.0557 (19)	0.0439 (18)	0.100 (2)	−0.0009 (15)	0.0019 (17)	−0.0026 (16)
C1	0.0459 (17)	0.0442 (17)	0.099 (2)	−0.0030 (14)	0.0063 (16)	0.0055 (16)
C12	0.0490 (18)	0.0501 (19)	0.095 (2)	0.0104 (15)	−0.0030 (16)	−0.0094 (16)
C8	0.060 (2)	0.0479 (19)	0.124 (3)	0.0022 (16)	0.015 (2)	0.0013 (19)
C2	0.0458 (18)	0.0491 (19)	0.126 (3)	0.0060 (15)	0.0120 (18)	0.0158 (19)

### Geometric parameters (Å, °)

**Table:**

N7—C13	1.328 (3)	C7—C9	1.497 (4)
N7—C14	1.337 (4)	C6—H6	0.9300
N2—C3	1.363 (4)	C10—H10	0.9300
N2—C1	1.378 (4)	N1—C3	1.294 (4)
N2—C4	1.410 (4)	N1—C2	1.367 (4)
N4—C4	1.325 (4)	C11—C12	1.344 (4)
N4—C7	1.342 (4)	C11—H11	0.9300
N8—C13	1.326 (3)	C9—H9A	0.9600
N8—C16	1.345 (3)	C9—H9B	0.9600
N6—C10	1.373 (4)	C9—H9C	0.9600
N6—C11	1.379 (4)	C3—H3	0.9300
N6—C13	1.413 (3)	C18—H18A	0.9600
C14—C15	1.379 (4)	C18—H18B	0.9600
C14—C17	1.500 (4)	C18—H18C	0.9600
N3—C4	1.321 (4)	C17—H17A	0.9600
N3—C5	1.347 (4)	C17—H17B	0.9600
C15—C16	1.375 (4)	C17—H17C	0.9600
C15—H15	0.9300	C1—C2	1.340 (5)
N5—C10	1.292 (4)	C1—H1	0.9300
N5—C12	1.375 (4)	C12—H12	0.9300
C5—C6	1.378 (4)	C8—H8A	0.9600
C5—C8	1.498 (4)	C8—H8B	0.9600
C16—C18	1.495 (4)	C8—H8C	0.9600
C7—C6	1.377 (4)	C2—H2	0.9300
			
C13—N7—C14	115.3 (2)	C12—C11—N6	105.5 (3)
C3—N2—C1	105.6 (2)	C12—C11—H11	127.3
C3—N2—C4	126.9 (3)	N6—C11—H11	127.3
C1—N2—C4	127.4 (2)	C7—C9—H9A	109.5
C4—N4—C7	115.4 (2)	C7—C9—H9B	109.5
C13—N8—C16	115.1 (2)	H9A—C9—H9B	109.5
C10—N6—C11	106.0 (2)	C7—C9—H9C	109.5
C10—N6—C13	126.9 (2)	H9A—C9—H9C	109.5
C11—N6—C13	127.0 (2)	H9B—C9—H9C	109.5
N8—C13—N7	128.9 (2)	N1—C3—N2	112.7 (3)
N8—C13—N6	115.4 (2)	N1—C3—H3	123.6
N7—C13—N6	115.6 (2)	N2—C3—H3	123.6
N7—C14—C15	120.7 (3)	C16—C18—H18A	109.5
N7—C14—C17	117.0 (3)	C16—C18—H18B	109.5
C15—C14—C17	122.3 (3)	H18A—C18—H18B	109.5
C4—N3—C5	114.8 (3)	C16—C18—H18C	109.5
C16—C15—C14	119.5 (3)	H18A—C18—H18C	109.5
C16—C15—H15	120.3	H18B—C18—H18C	109.5
C14—C15—H15	120.3	C14—C17—H17A	109.5
N3—C4—N4	129.1 (3)	C14—C17—H17B	109.5
N3—C4—N2	115.2 (2)	H17A—C17—H17B	109.5
N4—C4—N2	115.7 (2)	C14—C17—H17C	109.5
C10—N5—C12	105.0 (3)	H17A—C17—H17C	109.5
N3—C5—C6	120.9 (3)	H17B—C17—H17C	109.5
N3—C5—C8	116.8 (3)	C2—C1—N2	105.5 (3)
C6—C5—C8	122.4 (3)	C2—C1—H1	127.2
N8—C16—C15	120.6 (2)	N2—C1—H1	127.2
N8—C16—C18	116.9 (2)	C11—C12—N5	111.3 (3)
C15—C16—C18	122.6 (3)	C11—C12—H12	124.3
N4—C7—C6	120.5 (3)	N5—C12—H12	124.3
N4—C7—C9	116.9 (3)	C5—C8—H8A	109.5
C6—C7—C9	122.6 (3)	C5—C8—H8B	109.5
C7—C6—C5	119.2 (3)	H8A—C8—H8B	109.5
C7—C6—H6	120.4	C5—C8—H8C	109.5
C5—C6—H6	120.4	H8A—C8—H8C	109.5
N5—C10—N6	112.2 (3)	H8B—C8—H8C	109.5
N5—C10—H10	123.9	C1—C2—N1	111.6 (3)
N6—C10—H10	123.9	C1—C2—H2	124.2
C3—N1—C2	104.5 (3)	N1—C2—H2	124.2
			
C16—N8—C13—N7	−0.5 (4)	C13—N8—C16—C18	179.4 (3)
C16—N8—C13—N6	179.7 (2)	C14—C15—C16—N8	0.9 (4)
C14—N7—C13—N8	0.9 (4)	C14—C15—C16—C18	−178.9 (3)
C14—N7—C13—N6	−179.3 (2)	C4—N4—C7—C6	1.0 (4)
C10—N6—C13—N8	−175.0 (3)	C4—N4—C7—C9	−178.3 (2)
C11—N6—C13—N8	3.8 (4)	N4—C7—C6—C5	−2.2 (4)
C10—N6—C13—N7	5.2 (4)	C9—C7—C6—C5	177.1 (3)
C11—N6—C13—N7	−176.0 (3)	N3—C5—C6—C7	1.7 (4)
C13—N7—C14—C15	−0.3 (4)	C8—C5—C6—C7	−178.0 (3)
C13—N7—C14—C17	−179.9 (3)	C12—N5—C10—N6	0.7 (4)
N7—C14—C15—C16	−0.6 (4)	C11—N6—C10—N5	−0.5 (4)
C17—C14—C15—C16	179.0 (3)	C13—N6—C10—N5	178.5 (3)
C5—N3—C4—N4	−1.2 (4)	C10—N6—C11—C12	0.1 (3)
C5—N3—C4—N2	179.3 (2)	C13—N6—C11—C12	−178.9 (3)
C7—N4—C4—N3	0.8 (4)	C2—N1—C3—N2	−0.3 (4)
C7—N4—C4—N2	−179.7 (2)	C1—N2—C3—N1	0.3 (4)
C3—N2—C4—N3	2.8 (4)	C4—N2—C3—N1	178.3 (3)
C1—N2—C4—N3	−179.7 (3)	C3—N2—C1—C2	−0.2 (4)
C3—N2—C4—N4	−176.8 (3)	C4—N2—C1—C2	−178.1 (3)
C1—N2—C4—N4	0.8 (4)	N6—C11—C12—N5	0.3 (4)
C4—N3—C5—C6	−0.1 (4)	C10—N5—C12—C11	−0.6 (4)
C4—N3—C5—C8	179.6 (3)	N2—C1—C2—N1	0.0 (4)
C13—N8—C16—C15	−0.4 (4)	C3—N1—C2—C1	0.2 (4)
